# Mechanistic Insights Into Gut Microbiome Dysbiosis-Mediated Neuroimmune Dysregulation and Protein Misfolding and Clearance in the Pathogenesis of Chronic Neurodegenerative Disorders

**DOI:** 10.3389/fnins.2022.836605

**Published:** 2022-02-25

**Authors:** Piyush Padhi, Carter Worth, Gary Zenitsky, Huajun Jin, Kumar Sambamurti, Vellareddy Anantharam, Arthi Kanthasamy, Anumantha G. Kanthasamy

**Affiliations:** ^1^Parkinson’s Disorder Research Laboratory, Department of Biomedical Sciences, Iowa State University, Ames, IA, United States; ^2^Department of Physiology and Pharmacology, Center for Brain Sciences and Neurodegenerative Diseases, University of Georgia, Athens, GA, United States; ^3^Department of Neuroscience, Medical University of South Carolina, Charleston, SC, United States

**Keywords:** neurodegeneration, microbiome, neuroinflammation, protein aggregation, gut microbiota, gut metabolome, gut inflammation, Alzheimer’s and Parkinson’s diseases

## Abstract

The human gut microbiota is a complex, dynamic, and highly diverse community of microorganisms. Beginning as early as *in utero* fetal development and continuing through birth to late-stage adulthood, the crosstalk between the gut microbiome and brain is essential for modulating various metabolic, neurodevelopmental, and immune-related pathways. Conversely, microbial dysbiosis – defined as alterations in richness and relative abundances – of the gut is implicated in the pathogenesis of several chronic neurological and neurodegenerative disorders. Evidence from large-population cohort studies suggests that individuals with neurodegenerative conditions have an altered gut microbial composition as well as microbial and serum metabolomic profiles distinct from those in the healthy population. Dysbiosis is also linked to psychiatric and gastrointestinal complications – comorbidities often associated with the prodromal phase of Parkinson’s disease (PD) and Alzheimer’s disease (AD). Studies have identified potential mediators that link gut dysbiosis and neurological disorders. Recent findings have also elucidated the potential mechanisms of disease pathology in the enteric nervous system prior to the onset of neurodegeneration. This review highlights the functional pathways and mechanisms, particularly gut microbe-induced chronic inflammation, protein misfolding, propagation of disease-specific pathology, defective protein clearance, and autoimmune dysregulation, linking gut microbial dysbiosis and neurodegeneration. In addition, we also discuss how pathogenic transformation of microbial composition leads to increased endotoxin production and fewer beneficial metabolites, both of which could trigger immune cell activation and enteric neuronal dysfunction. These can further disrupt intestinal barrier permeability, aggravate the systemic pro-inflammatory state, impair blood–brain barrier permeability and recruit immune mediators leading to neuroinflammation and neurodegeneration. Continued biomedical advances in understanding the microbiota-gut-brain axis will extend the frontier of neurodegenerative disorders and enable the utilization of novel diagnostic and therapeutic strategies to mitigate the pathological burden of these diseases.

## Introduction

The harmonious symbiotic evolution of microbes is essential for normal neurodevelopment (Warner, 2019), immune maturation (Inlender et al., 2021), and protection against pathogens in humans and animals (Pickard et al., 2017). Microbes are detected in every exposed organ, including the skin, gastrointestinal (GI) tract, oral cavity, nares, and bronchial tracts. However, the greatest microbial species density and diversity are found in the GI tract. The number of GI microbes in humans is estimated at 10^13^–10^14,^ consisting primarily of various bacteria and lower amounts of archaea, fungi, and viruses (Sender et al., 2016). Astonishingly, the number of bacteria present in our body shows a nearly equal ratio to human cells (1.3:1). It boasts immense metabolic capabilities as the gut-microbiota may contain up to 23 million genes – a number that dwarfs the human genome (Claesson et al., 2009; Sender et al., 2016; Tierney et al., 2019). Since gut-residing bacteria are vital for host survival, understanding their influence can broaden the horizon for diagnosis and therapy for complex multifactorial diseases, including neurodegenerative diseases.

The human gut microbiome is primarily composed of two dominant major bacterial phyla, Bacteroidetes and Firmicutes, composing 90% of the total gut bacteria with the remaining 10% represented by other phyla including Actinobacteria, Proteobacteria, Fusobacteria, and Verrucomicrobia. Despite a predominant pattern that emerges among the higher taxonomic categories, the gut microbiota is highly dynamic and individualized based on a person’s early life composition, diet, exercise, lifestyle factors, and disease status (Matsumoto et al., 2008; Desai et al., 2016; Martin et al., 2016; Xu et al., 2019). Furthermore, the regional and temporal variations influence microbial diversity and the host’s response to disease states (Arumugam et al., 2011). The microbial composition diversifies from *in utero* to early and mid-stage adulthood, followed by a progressive collapse of healthy microbiota in late adulthood (Lahiri et al., 2009; Martinez et al., 2018; Stinson et al., 2019; Yang et al., 2019; Mitchell et al., 2020). Although the existence of a placental microbiome during the *in utero* stage of development is contentious, evidence suggests the presence of bacteria and short-chain fatty acids (SCFAs) in the meconium and amniotic fluid (Stinson et al., 2019). Furthermore, at birth, the infant’s microbiome is contingent on the mode of delivery (i.e., birth canal or Cesarean delivery). The mother’s skin or vaginal microbiome signature is imprinted, enabling early colonizers to shape the infant’s long-term microbial composition, diversity, and early immune development (Martinez et al., 2018; Yang et al., 2019; Mitchell et al., 2020). Aging, a major risk factor in the pathological progression of neurodegenerative disorders, is also associated with modulating the gut microbial ecosystem (Bosco and Noti, 2021). It contributes to a compositional shift in gut microbiota characterized by a dramatic decrease in the diversity and abundance of several beneficial bacteria (reviewed in Dinan and Cryan, 2017). Additionally, the host’s genetics and environmental factors such as diet or exposure to toxins can further augment disease states (reviewed in Cabrera et al., 2021). A classic example of diet-induced gut dysbiosis was recorded in the early 70’s, where individuals from southern India who migrated to the United Kingdom in the 1970s frequently suffered from severe vitamin B12 deficiency (Britt et al., 1970, 1971; Stewart et al., 1970; Roberts et al., 1973; Britt and Harper, 1976). The subjects who presented with normal Vitamin B12 levels were found to consume a diet similar to that of South India and a later investigation reported that the synthesis of vitamin B12 precursors was modulated by microbial flora in the small intestine (Mathan et al., 1974; Albert et al., 1980). Since then, it has become evidently clear from several pre-clinical animal and human cohort studies that the microbiome is crucial for shaping various metabolic, immunological, and neurodevelopmental homeostatic processes (De Vadder et al., 2018; Rothhammer et al., 2018; Wang et al., 2020), and that cumulative exposure to infections, poor diet, and antibiotics can trigger dysbiosis and exacerbate the progression of several chronic age-related disorders (reviewed in Soto et al., 2018; Fang et al., 2020; Bosco and Noti, 2021).

**FIGURE 1:**
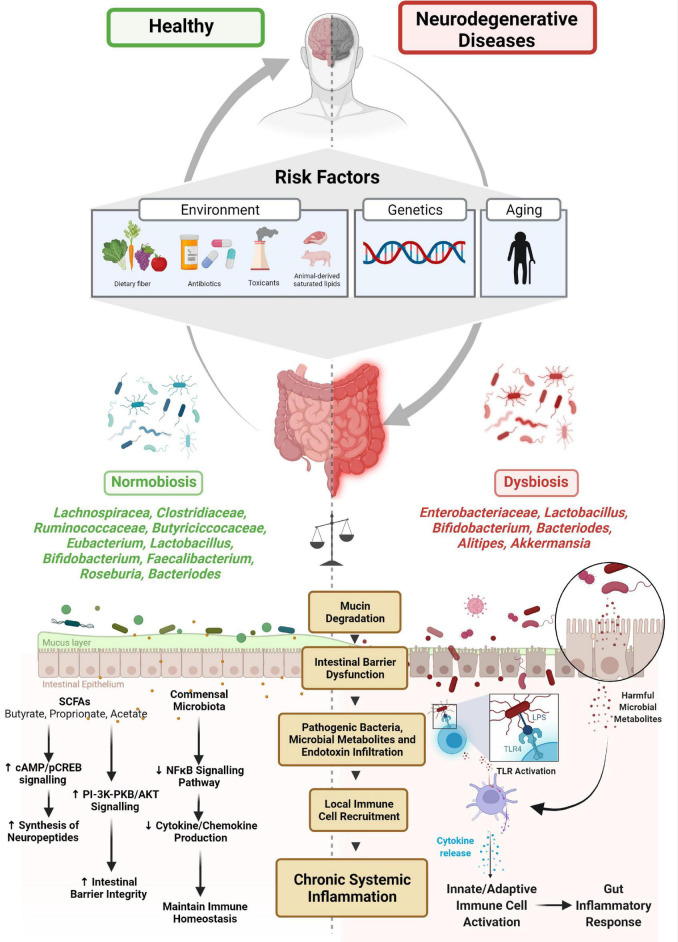
Microbiome-gut-brain axis and neurodegenerative disease. Several risk factors have been associated with the etiology of microbial dysbiosis and neurodegenerative disorders. The balance between normobiosis and dysbiosis within the gut microbiome is preserved by the presence or absence of high-abundant species (*Lachnospiracea, Clostridiaceae, Ruminococcaceae, Eubacterium, Butyriciccocaceae, Lactobacillus, Bifidobacterium, Faecalibacterium, Roseburia*, and *Bacteroides*) and low-abundant species (*Enterobacteriaceae, Alistipes*, and *Akkermansia*). In the presence of beneficial microbes, homeostatic mechanisms such as neuropeptide synthesis, promotion of intestinal barrier integrity and immune cell regulatory functions are maintained. Higher abundances of microbes are associated with dysbiosis, degrade mucin, dysregulate intestinal barrier, and enable pathogenic microbes and their metabolites and endotoxins to infiltrate, promoting local immune cell recruitment and triggering systemic inflammation.

The symbiotic and competitive relationships between diverse bacterial communities and hosts are preserved by enteric networks spanning various gut environments, and the central nervous system (CNS). The microbiota establishes a bi-directional communication network with the brain *via* neural, endocrine, and metabolic signaling modalities, hence the popular reference to the microbiome-gut-brain axis (Figure 1). A dysfunctional gut environment and its implications in nervous system disorders have been known for over two decades. More recently, a growing number of associative and mechanistic studies in neurobehavior, inflammation, neurogenesis, and neurodegeneration have implicated gut-residing microbes as key modulators of several disease etiologies (Soto et al., 2018; Gershon, 2019; Ma et al., 2019; Fang et al., 2020). Specifically, in neurodegenerative disorders such as Parkinson’s disease (PD) and Alzheimer’s disease (AD), recent findings indicate that the detrimental alterations to microbial composition augment disease-specific pathology. This review aims to provide some mechanistic insights into how a shift in specific populations of microbes and their derived metabolites in the gut triggers and facilitates various disease processes, including chronic intestinal and systemic inflammation, protein misfolding and aggregation, dysfunctional protein clearance, and a heightened autoimmune response, thereby contributing to disease propagation in the brain. This review also discusses how the microbiota influences the enteric nervous system (ENS) and CNS and modulates neuro-immune synapses and enteric neuropathy.

## Understanding Gut Microbial Dysbiosis in the Context of Neurodegenerative Disorders – A Metagenomics Perspective

Advances in genomic technologies allowed for massive cataloging of genomes from fecal microbes to be directly extracted and evaluated by gene ontology bioinformatics tools to understand the biological processes of the human-microbe hybrid (Backhed et al., 2005; Gill et al., 2006). Studies on the role of microbes in health were fostered by the setting up of National Institutes of Health (NIH)- and European-sponsored human microbiome projects that developed publicly available databases of healthy microbiota (see NIH-Human Microbiome Project and European MetaHIT consortium).

Microbial ecosystems are driven by the composition of distinct groups and subgroups of bacterial taxa cohabitating and interacting in a preferred community (Peterson et al., 2009; Arumugam et al., 2011). Using fecal metagenomic sequencing and 16S ribosomal RNA gene profiling from individuals from three continents, two landmark studies in 2011 and 2018 stratified the human gut microbiome into three distinct clusters, called Enterotypes 1 to 3 (Arumugam et al., 2011; Costea et al., 2018), each classified by a dominant genus: *Bacteroides* (Enterotype 1) and *Prevotella* (Enterotype 2) and a third Firmicutes dominant cluster with *Ruminococcus* (Enterotype 3) (Arumugam et al., 2011; Costea et al., 2018). More importantly, along with the dominant genera, a shared network of several co-occurring genera with similar functional properties was detected. For example, *Bacteroides* and its co-occurring enriched genera *Parabacteroides* share saccharolytic and proteolytic pathways, thereby deriving energy from diets enriched with animal protein fats and carbohydrate-rich foods. The functional attributes between the ‘microbial-cluster profiles’ were unique, varying in diversity, richness, and temporal stability, thereby supporting the notion that microbes exist in ecological niches within a particular gut environment, and cohabitating microbes share functionally similar metabolic environments (Costea et al., 2018; Jansma and El Aidy, 2021). Furthermore, cluster analyses revealed that the dominant genera with their co-occurring highly abundant microbes influence more macro-molecular functions (e.g., energy production pathways), and the less abundant microbial species detected in all three clusters supported the dominant genera through their involvement in more specialized functions (e.g., archaeon *Methanobrevibacter*, hydrogen producer and methanogen, and *Desulfovibrio*, a sulfate reducer) (Arumugam et al., 2011; Costea et al., 2018). Herein, high- and low-abundant species will be used to denote microbes with macro-molecular and specialized functions, respectively. The normobiosis of the gut ecosystem depends on a critical balance among the high- and low-abundant species and the degree to which selective pressures (e.g., environmental factors, genetic predisposition, and age of host) impact compositional features (Figure 1). It is evident from a growing number of studies that high-fat diets, drugs (antibiotics), and toxicants induce dysbiosis and improve the survivability of normally uncommon gut microbial species, leading to enhanced functional responses, pathogen infiltration, and further deterioration of gut health (Desai et al., 2016). Therefore, a detailed network mapping of high- and low-abundant, cohabitating microbes can generate ‘microbial-cluster profiles’ that provide a dynamic view of microbe-microbe and microbe-host interactions and help stratify the heterogeneous multifactorial disease types (Vascellari et al., 2021). Second, cluster analyses can aid in clinical subtyping while also identifying and diagnosing microbe-derived gene signatures as potential biomarkers for neurodegenerative disease (Qian et al., 2020). And finally, results obtained from meta-analyses from clinical data can be corroborated with a more ‘fine-tuned’ mechanistic evaluation in animal models to unravel microbe-mediated pathophysiological changes (Romano et al., 2021).

## Microbiota-Enteric-Gut-Brain Axis

Unlike other organ systems, the gut is under a constant state of inflammation, observed by a huge expanse of lymphocytes in the lamina propria and the intra-epithelial compartment of the small and large intestines (reviewed in Powell et al., 2017). It can defend from various pervasive pathogens while immunologically tolerating beneficial commensal microbes, densely distributed across a vast, convoluted surface area. Along with the presence of high- and low-abundant microbes, the maintenance of the intestinal epithelial barrier is further maintained by critical players, including immune cells (mononuclear macrophages, T-cells, and innate lymphoid cells), enteric glial cells (EGCs), and the intrinsic/extrinsic enteric-associated-neurons (iEANs/eEANs) of the ENS (Gabanyi et al., 2016; Gury-BenAri et al., 2016; Chen et al., 2018; Jarret et al., 2020; Muller et al., 2020b). The functions of such vital players have been reviewed by Powell et al. (2017) and Natale et al. (2021) and will not be addressed in this review. Given the knowledge gaps in understanding the etiology of neurodegenerative disease, emerging research in microbe-dependent modulation of the ENS and the microbe-ENS-brain axis provides clues into the emergence of enteric dysfunction and neurodegenerative diseases.

### The Microbiota – A Modulator of Gut Physiology

The ENS encompasses vast networks of extrinsic and intrinsic neuronal circuits that independently control endocrine functions, conduct transmucosal fluid movement, and regulate local blood flow and motility *via* reflex systems (reviewed in Rao and Gershon, 2016). Moreover, its intimate connection with gut microbiota is crucial for enteric neurogenesis and neuroprotection (De Vadder et al., 2018), while its colonization is essential for neuronal cell population density (Collins et al., 2014) and neuron-dependent motility reflexes (Delungahawatta et al., 2017). Depleting microbes by antibiotics reduces motility reflexes *ex vivo* (Delungahawatta et al., 2017), alters colonic motility and neurochemicals, and disrupts enteric neuronal networks in post-weaned animals (Hung et al., 2020). Similarly, a high-fat diet elicits microbial dysbiosis in juvenile rats, myenteric neuronal loss and decreased nitrogenic neurons (McMenamin et al., 2018). In addition to the enteric neurons, microbial colonization enables early postnatal EGC maturation (Inlender et al., 2021) and influences renewal and preservation for the enteric glial population (Kabouridis et al., 2015). Interestingly, veterans of the Persian Gulf War experience GI disorders similar to those of irritable bowl syndrome (IBS) and irritable bowel disease (IBD) (Koch and Emory, 2005). Studies in animal models of Gulf War Syndrome suggest gut-microbial dysbiosis likely increases immune activation mediated by the TLR4-S100β/RAGE-iNOS pathway of EGCs followed by redox instability and dysfunction of the GI barrier (Kimono et al., 2019), further implicating the microbiota as an important regulator of enteric neuronal and glial physiology, independent of CNS modulation.

Although many ENS functions are independent of CNS input, the ENS does function as an interface to detect intestinal luminal cues that trigger upstream CNS signaling pathways. In a landmark paper from *Nature*, Muller et al. (2020b) identified eEANs in contact with intestinal epithelial cells can detect microbes and their metabolites, specifically SCFAs, bile acids, and neuropeptides, *via* vagal afferents and subsequently sympathetically modulate gut activity *via* a CNS-directed circuit (Muller et al., 2020b). Upon loss of microbial signals, modeled by germ-free, gnotobiotic, and broad-spectrum antibiotic-treated rodents, eEANs sensed the changes in intestinal epithelial and mucosal areas in the lamina propria, relayed signals to sensory vagal afferents of the nodose ganglion and dorsal vagal complex, sympathetically activated glutaminergic neurons lateral to the paragigantocellular nucleus/rostral ventrolateral medulla (LPGi/RVLM) and downstream coeliac-superior mesenteric ganglia (CM-SMG), whereby efferent nerves reduced GI transit time. As this study comprehensively evaluated how microbes and their derived metabolites alter gut physiology by direct CNS-mediated gut sympathetic modulation, separate research implicates the role of the aryl hydrocarbon receptor (AHR) as a critical node in regulating gut physiology and neurogenesis (Obata et al., 2020; Wei et al., 2021). AHR, a transcriptional factor differentially expressed in various intestinal segments and dominant in colonic enteric neurons, integrates the signals from the luminal environment and regulates intestinal peristalsis upon microbial colonization (Obata et al., 2020). Furthermore, AHR signaling was also relevant in intestinal barrier function and the mucosal immune system (Metidji et al., 2018; Rothhammer and Quintana, 2019), further implicating its role as a regulatory node, central to maintaining gut homeostasis and a possible biomarker for GI disorders (Obata et al., 2020). Beyond the ENS, this transcriptional factor when selectively activated by the microbial metabolite indole produced by tryptophanase-expressing gut microbes interestingly induces neurogenic effects in the adult murine hippocampus as identified by elevated expression of β-catenin, Neurog2, and VEGF-α (Wei et al., 2021). While administration of antibiotics decreases adult neurogenesis (Mohle et al., 2016), the indole-AHR signaling pathway highlights microbiota as a direct modulator to hippocampal neurogenesis.

### The Microbial Neuro-Immune Synapse

In addition to the sensory afferents and autonomic efferents of the extrinsic enteric system, the eEANs concurrently communicate and transmit information to the iEANs (Matheis et al., 2020). However, iEANs exhibit a more region-specific anatomical and gene expression profile than do eEANs, indicating their distinct functional role in maintaining GI functions. Recent evidence signifies the importance of microbes in regulating iEAN-mediated GI homeostasis (Matheis et al., 2020). As the diversity and abundance of microbes increase from the upper GI to distal sites, the number of iEANs likewise increases. The total cell count, morphology, and the gene expression profile of iEANs in the duodenum, ileum, and colonic myenteric plexus varies significantly, with a higher neuronal count in specific-pathogen-free mice compared to germ-free animals (Matheis et al., 2020). GI physiology necessitates the presence of commensal microbes. Compared to both germ-free and antibiotic-administered rodents, commensal microbiota lead to an increased synthesis of neuropeptides, measured indirectly by phosphorylated cAMP response element-binding protein (pCREB), neuropeptide transcripts and protein expression of somatostatin (SST) and cocaine- and amphetamine-regulated transcript (CART) in the ileum and colon. Additionally, the absence of microbiota, or the presence of broad-spectrum antibiotic-induced dysbiosis, triggered the NOD-like receptor family pyrin domain (PYD)-containing 6 (NLRP6)/Caspase 11 (Casp11) inflammasome-mediated pathway and subsequent loss of iEANs and enteric neuropathy (Muller et al., 2020a). Critically, this is especially relevant to GI-mediated complications commonly associated with neurodegenerative disorders, in which GI motility impairment, chronic low-grade inflammation, and intestinal nerve damage are widely observed (De Giorgio et al., 2004; Chalazonitis and Rao, 2018). Like neuron-glia interaction in the CNS, the ENS forms a ‘neuro-immune synapse’ with its local immune players to limit enteric neuropathy and maintain gut health. Recent evidence suggests that intestinal resident muscularis macrophages directly form synapses with iEANs and play an essential role in limiting iEAN neuropathy and mediating their pathogen-resistance mechanism (Matheis et al., 2020). Unlike the lamina propria-residing macrophages, which sense and respond *via* pathogen-induced phagocytotic mechanisms to clear dying or senescent intestinal epithelial cells, muscularis macrophages present a tissue-protective phenotype by catecholamine-mediated activation of the β2-adrenergic receptor signaling pathway, triggered upon enteric infection. This further highlights the essential role of microbial composition in altering gut-pathogen resistance mechanisms by sympathetic modulation at the neuro-immune synaptic junctions (De Schepper et al., 2018).

## Gut Microbiome and Neurodegenerative Diseases

### Parkinson’s Disease

Parkinson’s disease is a heterogeneous disease with multiple subtypes (Chaudhuri et al., 2006; Lawton et al., 2018). However, before the onset of clinically diagnosable motor symptoms, most patients experience several non-motor symptoms, including depression, apathy, dementia, rapid eye movement (REM), sleep behavior disorder (RBD), and GI-associated disorders, including reduced gastric emptying and constipation (Chaudhuri et al., 2006; Seppi et al., 2011; Fasano et al., 2015). Although still controversial, several lines of evidence from metagenomic studies of PD patients and healthy age-matched individuals suggest dysbiosis within the gut microbiome of PD patients could modify the risk and progressively worsen disease status (Sampson et al., 2016; Qian et al., 2020; Romano et al., 2021). Despite methodological differences across studies, including inclusion criterion, sample collection, disease status, or additional confounders, contributing to divergent microbial profiles, the evidence of microbial dysbiosis in PD is consistent (Romano et al., 2021; Rosario et al., 2021). Individuals with PD have dramatically divergent microbial profiles compared to healthy controls (Romano et al., 2021). Dominant taxa (e.g., *Lachnospiraceae*, *Ruminococcaceae*, *Faecalibacterium, Roseburia*, and *Butyriciccocaceae*) that are part of the core microbial community specializing in carbohydrate and energy metabolism and involved in the production of butyrate and other SCFAs, decrease in PD patients, while the genera *Akkermansia, Lactobacillus*, and *Bifidobacterium* increase (Geirnaert et al., 2014; Vacca et al., 2020). Although *Lactobacillus* and *Bifidobacterium* spp. are considered probiotics and often associated with improving constipation in PD, it is unclear whether their increased abundance is due to a transient immune compensatory response in already immune-compromised patients (Suez et al., 2019; Tan et al., 2021).

As dietary fiber is vital for maintaining the colonic mucus barrier, fiber deficiency favors the proliferation of distinct microbial populations that degrade the colonic mucus layer and enable enhanced colonization and infiltration of opportunistic pathogens (Desai et al., 2016). *Akkermansia* spp., relatively sparse in healthy subjects, are consistently enriched in PD samples versus controls (Hill-Burns et al., 2017; Wallen et al., 2020; Rosario et al., 2021) and are increasingly abundant in the fecal microbiome of patients who experience constipation, a primary non-motor symptom of PD. Nishiwaki et al. (2020) implicated this genus in the neuropathological progression of PD by mechanisms of degrading mucin. Mucin degradation and altered intestinal *O*-glycans expression contribute to the eventual erosion of colonic mucosal layers, identified by decreased periodic acid-Schiff (PAS)-positive goblet cells, which could compromise the intestinal barrier (An et al., 2007; Cao et al., 2017) and increase susceptibility to pathogenic infiltration (Desai et al., 2016) (Figure 1). Indeed, an increased abundance of *Akkermansia* in PD individuals is correlated with an increased presence of co-occurring opportunistic pathogens (Wallen et al., 2020), thereby supporting the notion that higher levels of typically less common microbial species represent a risk factor in modifying PD pathophysiology (Wallen et al., 2020).

### Alzheimer’s Disease

Like PD, the influence of microbial dysfunction is further implicated in the etiopathogenesis of AD. Although variability in compositional features exists in separate clinical cohort studies, AD patients demonstrate a differential abundance of several genera, mainly fewer *Lachnospiraceae*, *Clostridiaceae* and *Ruminococcaceae*, and *Eubacterium*, with more *Bacteroides*, *Bifidobacterium, Enterobacteriaceae*, *Alistipes*, and *Akkermansia* taxa (Vogt et al., 2017; Liu et al., 2019; Ling et al., 2020; Marizzoni et al., 2020; Lee et al., 2021b; Nara et al., 2021). Furthermore, selected genera such as *Escherichia/Shigella* isolated from participants’ stool samples have been shown to directly trigger a pro-inflammatory state and amyloid-β accumulation (Cattaneo et al., 2017). Amyloid deposition and peripheral inflammatory mediators are commonly linked to AD pathophysiology. The involvement of gut microbiota in triggering neuroinflammatory pathways was previously observed in antibiotic-treated rodents (Minter et al., 2016). In the APP (Swe)/[PS1(L166P)] transgenic (Tg) model of AD, germ-free status reduced cerebral Aβ load compared to conventionally raised APP rodents (Harach et al., 2017), implicating microbiota as a direct or indirect modifier of peripheral amyloidosis (discussed later in Section “Gut Microbiome-Mediated Fibril Formation”). Functional analyses of the gut microbiota in AD patients reveal broad metabolic changes, including alterations in bacterial cell motility, lipoic acid metabolism, and glycan degradation (Ling et al., 2020). How global functional changes are implicated in the pathophysiology of AD is yet to be uncovered. Specific bacterial taxa, such as *Bacteriodetes* spp., is associated with AD dementia. However, reports are conflicting as some suggest it could increase the risk of dementia, while others propose the opposite effect (Alkasir et al., 2017; Vogt et al., 2017; Saji et al., 2020). *Bacteriodetes* are common Gram-negative bacteria commensal to the human gut and are responsible for producing SCFAs in specific GI environments (Parada Venegas et al., 2019). However, in cases of chronic microbial dysbiosis induced by exposure to environmental toxicants, ingestion of a high-fat diet can cause changes in the GI environment that can enable distinct microbes to proliferate and infiltrate neighboring gut environments, and subsequently trigger deleterious inflammatory pathways (Figure 1) (Sun et al., 2018; Miao et al., 2021). Certain species within *Bacteriodetes* taxa, such as *Bacteroides fragilis*, contain lipopolysaccharide (LPS) in its outer membrane, and the toxin fragilysin, a pro-inflammatory zinc metalloprotease, can trigger systemic inflammation and amyloid fibrillogenesis (Lukiw, 2016; Zhao et al., 2017). Since aging is a primary risk factor in neurodegenerative diseases, it is likely that the age-dependent reduction in microbial species diversity, including alterations in relative abundances of region-specific microbes, further facilitates persistent degradation and damage of gut mucosal and gut epithelial layers and increases infiltrations of bacterial toxins (Qin et al., 2007; Peniche et al., 2018; Xu et al., 2019). Hyper-stimulation of low-grade inflammatory mediators triggers peripheral endotoxemia and a persistent inflammatory state, popularly called “inflammaging” (reviewed in Franceschi et al., 2018; Brown, 2019; Kowalski and Mulak, 2019; Jukic Peladic et al., 2021).

## Mechanistic Insight Into Gut Microbiome-Mediated Neurodegeneration

A clear pathophysiological link between immune and metabolic dysregulation and neurodegenerative disorders in the brain had been previously established (reviewed in Hammond et al., 2019; Yan et al., 2020). More recently, evaluation of microbiome-mediated gut dysregulation provides insight into the peripheral etiology of neurodegenerative disorders such as PD and AD.

### Chronic Gut-Inflammation and Intestinal Barrier Dysfunction Impact the Status of Neurodegenerative Disorders – Relevance to Irritable Bowel Disease

Chronic GI inflammation increases the risk of PD (Lee et al., 2021a) and AD-dementia (Zhang et al., 2021). Emerging studies suggest that the enteric microbiome – neuroimmune system interaction could contribute to a novel etiopathogenesis of neurodegenerative disorders. Of the GI-associated conditions commonly associated with an increased risk of neurodegenerative disease, IBS (Liu et al., 2021) and IBD are the most relevant; however, this section will focus on IBD (Kim et al., 2021). The genetic and pathophysiological overlap between IBD and neurodegenerative diseases converges on shared mechanisms, representing early therapy targets.

Recent epidemiological evidence suggests that an increased incidence of IBD is associated with a 20–90% higher risk of PD (Lee et al., 2021a). Furthermore, younger IBD patients aged 40–65 years are at greater risk of developing PD (Kim et al., 2021); however, individuals already with PD are not at risk of developing IBD (Lee et al., 2021a). If chronic gut inflammation can trigger the onset of PD, perhaps early intervention strategies could prevent or slow the downstream pathology. Indeed, in a retrospective study, administration of anti-tumor necrosis factor (anti-TNF) to clinically diagnosed IBD patients led to a 78% reduction in the risk of developing PD, compared to untreated controls (Peter et al., 2018). A similar reduction was observed with the administration of other anti-inflammatory agents, including 5-aminosalicylic acid, azathioprine, and corticosteroids (Park et al., 2019; Pinel Rios et al., 2019). It is evident that, like in IBD, intestinal inflammation and intestinal barrier dysfunction are similarly apparent in PD. Increased inflammatory markers such as CD8B and NFκB p65 were identified in colon biopsies in PD patients, with reduced expression of the regulator protein G-protein signaling 10 (RSG10) in peripheral immune cells (Houser et al., 2021). RGS10 is expressed in myeloid-cell lines and is involved in the neuroprotective mechanism of inhibiting NFκB activity (Lee et al., 2011). RGS10 deficiency triggered intestinal inflammation in RGS10-knockout (KO) mice and augmented MPTP-induced nigrostriatal dopaminergic degeneration (Houser et al., 2021). The occurrence of morphological and functional alterations in the intestinal epithelial layer of PD patients is also evidenced by increased alpha-1-antitrypsin and zonulin in human fecal samples (van IJzendoorn and Derkinderen, 2019; Aho et al., 2021) and the reduction of zona occludens proteins (ZO-1) as revealed by immunofluorescence staining (Kuan et al., 2016). The gut microbiota plays a critical role in maintaining the mucosal intestinal barrier, and exposure to environmental toxicants can further exacerbate PD phenotype (Dodiya et al., 2020). Chronic administration of rotenone, a potent well-studied toxicant of PD, triggered tyrosine hydroxylase-positive neuronal loss in the substantia nigra in both conventionally raised animals (Kanthasamy et al., 2019; Bhattarai et al., 2021) and germ-free animals (Bhattarai et al., 2021). However, decreased motor control and motor coordination as well as disruption of the intestinal epithelial barrier were observed in rotenone-treated conventionally raised mice, but not in rotenone-administered germ-free mice, thereby linking the gut microbiota to PD etiology (Bhattarai et al., 2021). Intriguingly, an altered gut microbiota, a defective intestinal barrier, and gut inflammation were similarly observed in the MPTP mouse model of PD. Timed, subacute MPTP administration disrupted the intestinal barrier, immune status, and gut microbiota after the first dose. These effects recovered; however, the second MPTP dose further exacerbated gut dysbiosis and gut barrier dysfunction, possibly further implicating the microbiota as a modifier of developing PD (Xie et al., 2020).

Beneficial bacteria such as the genera *Ruminococcaceae* and *Lachnospiraceae* play an essential role in strengthening the intestinal barrier by producing SCFAs (Baxter et al., 2019). PD patients consistently display lower abundances of these microbes, thereby significantly lowering concentrations of SCFAs, including acetate, propionate, and butyrate (Aho et al., 2021; Baert et al., 2021). As these critical dietary metabolites were previously discussed to regulate the mucosal immune system and protect gut epithelial layers in IBD (Parada Venegas et al., 2019) and NDs (Pellegrini et al., 2018; Wang et al., 2020), a recent report suggests that propionate increases the expression of ZO-1 and occludin, thereby improving intestinal epithelial integrity by a serine-threonine kinase (AKT) signaling pathway (Huang et al., 2021). Additionally, certain conventionally dominant commensal bacteria like *Faecalibacterium prausnitzii*, which plays a critical role in maintaining immune homeostasis by inhibiting the NFκB signaling pathway and cytokine production and increasing colonic epithelial tight junction proteins, is significantly reduced in both IBD and PD (Laval et al., 2015; Lopez-Siles et al., 2016; Pellegrini et al., 2018). Therefore, a shift in the community of beneficial microbes decreases anti-inflammatory microbial mediators, thereby contributing to a persistent loss in gut barrier integrity and a constipation phenotype often noted in PD patients. Grun et al. (2020) reported that commonly prescribed PD drugs such as COMT inhibitors can further lower the abundance of *F. prausnitzii* and SCFAs concentrations, thereby enhancing the drug’s bioactivity and toxicity and further damaging the gut epithelial barrier leading to infiltration of other opportunistic pathogens (reviewed in Weersma et al., 2020).

In addition to exposure to environmental toxicants, several genetic risk factors are shared among IBD and PD patients (*ROH3P*, *HLA*, *CCNY*, *LRRK2*, *MAPT*, *SYMPK*, *RSPH6A*, *GUCY1A3*, *HLA*, *BTNL2*, and *TRIM10*). The most significant is the Leucine-rich-repeat kinase 2 (LRRK2), a multimeric protein with both kinase and GTPase activity associated with autosomal dominant PD. A missense mutation (G2019S) within *LRRK2* modifies α-synuclein pathology in a mouse PD model and increases kinase GTPase activity to inhibit autophagy and augment the pro-inflammatory response in mouse models of colitis (Takagawa et al., 2018; Bieri et al., 2019). Similarly, an autosomal recessive gene associated with early Parkinsonism, DJ-1 (PARK 7), is dysfunctional in human colonic tissue samples as well as in *in vitro* and *in vivo* experimental models of colitis (Zhang et al., 2020). As both IBD and PD have compromised intestinal barrier functions, DJ-1 deficiency promotes cytokine-mediated inflammation (IL-1β, IL-6, TNFα, and TGFB1) and apoptosis of intestinal epithelial cells *via* a p53-dependent mechanism *in vitro*, in *ex vivo* colonic sacs, and *in vivo* (van IJzendoorn and Derkinderen, 2019; Zhang et al., 2020; Lippai et al., 2021). This further supports the hypothesis that the onset, progression and severity of IBD and PD are aggravated by gene-environment interactions occurring at both the host and gut microbiome levels.

Commonalities also exist between IBD incidence and increased risk of AD. A recent Korean cohort study identified that IBD patients ≥65 years suffer an increased risk of AD compared to controls (Kim et al., 2021). These findings further supported a Taiwanese cohort study suggesting the overall incidence of AD dementia is significantly elevated among patients with IBD (Zhang et al., 2021). However, unlike PD, the average age of patients with IBD with dementia was ≥65 years, thereby enabling the possibility of future research investigating the functional consequence of an age-dependent reduction in commensal microbial diversity and richness on AD-dementia. In a recent *in vivo* study utilizing an aging mouse model of IBD, chronic colitis triggered spatial and memory deficits (He et al., 2021). Dextran sulfate sodium (DSS)-induced colitis caused amyloid plaque accumulation, compromised glymphatic clearance and increased infiltration of gut-derived T-cells, and triggered cortical and hippocampal degeneration mediated by NACHT-LRR and PYD-containing protein 3 (NLRP3) inflammasome expression (He et al., 2021). The absence of NLRP3 protected against neuroinflammatory and neurodegenerative processes in DSS-induced colitis in aged animals. Although a defined mechanistic evaluation of microbe-dependent pathology of AD-dementia is yet to be uncovered, previous studies indicate that AD pathogenesis is perhaps associated with age-dependent depletion of healthy microbes with concurrently increased burden of infectious opportune, enterotoxigenic bacteria and viruses, i.e., *Bacteroides fragilis* and herpes simplex virus-1 (HSV-1) (Desai et al., 2016; Lukiw et al., 2021). Both *B. fragilis* and HSV-1 are commensal to the human host and play a critical role in stimulating the innate immune response by the NFκB – microRNA-146a signaling pathway (Li et al., 2020). HSV-1 is noted to trigger peripheral infection and is relevant in the progression of AD (reviewed in McManus and Heneka, 2017; Zhao and Lukiw, 2018). As mentioned earlier, an enterotoxigenic form of *B. fragilis* can release a unique pro-inflammatory LPS-subtype and fragilysin, leading to an impaired intestinal paracellular and transcellular epithelial barrier, thereby allowing an already ‘leaky’ barrier a gateway for the enhanced entry of microbiome-derived neuro- and entero-toxins into the circulation (Lukiw et al., 2021). Enterotoxigenic *B. fragilis* triggered GI inflammation in colonic epithelial cells *via* G-protein coupled receptor-35 (GPCR35) binding. Colitis triggered in mouse models by enterotoxigenic *B. fragilis* signals bound to GPCR35 was reversed by a GPCR35 antagonist (Boleij et al., 2021).

MicroRNAs (miRNA) are single-stranded, short RNAs that post-transcriptionally regulate gene expression by binding to the 3′ untranslated region (UTR) and silencing the target genes (Chopra et al., 2020; Li et al., 2020). They can be detected in the brain, biofluids [urine, cerebrospinal fluid (CSF), and blood], and, more recently, in stool samples (Tarallo et al., 2021). With a growing number of studies elucidating the role of miRNAs in various disease processes, the gut-residing microbes can further modulate miRNA expression and regulate host pathophysiology. Exciting work by Cao et al. (2021) demonstrated that enterotoxigenic *B. fragilis* downregulated METTL14-mediated *N*^6^-methyladenosine (METTL14/m^6^A) methylation, thereby blocking the processing of miRNA-149-3p (miR-149-3p). The downregulation of exosome-derived miR-149-3p contributed to T-helper type 17 (Th17) cell differentiation, promoting intestinal inflammation (Cao et al., 2021). Plasma exosomal miR-149-3p was similarly reduced in IBD patients, implying miRNA is a potential biomarker for systemic immune dysregulation. As a growing number of studies implicate distinct microbial species in triggering intestinal inflammation, further mechanistic exploration is necessary to prove causality in neurodegenerative diseases.

### Gut Microbiome-Mediated Fibril Formation

**FIGURE 2:**
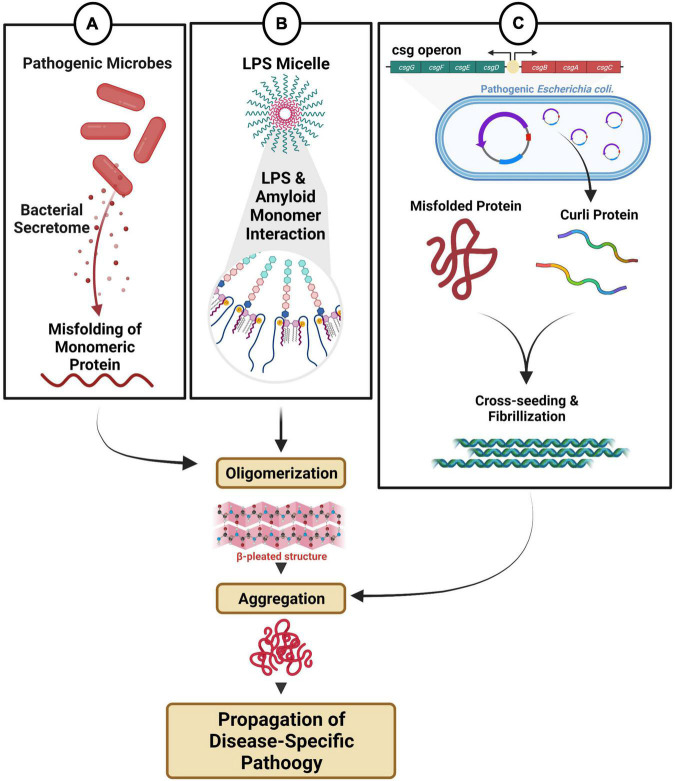
Microbial dysbiosis-mediated fibril formation. **(A)** Distinct microbes secrete metabolites that can directly and/or indirectly modulate fibril formation. **(B)** Endotoxin and LPS micelles can interact with amyloid monomeric protein triggering oligomerization and aggregation. **(C)** Pathogenic bacteria with csgA/B operon produce amyloid curli proteins, which can participate in cross-seeding, fibrillization and aggregation.

In PD, α-synuclein inclusion bodies evidenced in the brainstem, locus coeruleus, and dorsal motor nucleus of the vagus (DMV), were also present in the stomach, duodenum, colon (Shannon et al., 2012; Sanchez-Ferro et al., 2015), and enteric submucosal Meissner’s plexus (Kupsky et al., 1987; Wakabayashi et al., 1988; Braak et al., 2006) in both autopsied PD patients and biopsy samples of the prodromal stage of PD (Shannon et al., 2012; Hilton et al., 2014). Although the interaction between genetic risk factors and environmental toxins likely initiates the oligomerization, aggregation, and propagation of α-synuclein, gut microbiota-derived products can potentiate the pathologic process and further modify disease biology. When the fecal microbiota from PD patients was transplanted to an α-synuclein overexpression (ASO) mouse model, the mice displayed exacerbated α-synuclein inclusion bodies and PD-motor and GI deficits compared to mice transplanted with the microbiome from healthy donors (Sampson et al., 2016, 2020; Hill-Burns et al., 2017). Since a complex microbiota was found to be necessary for worsening α-synuclein pathology, it is likely that a combination of the increase in harmful bacteria, decrease in beneficial bacteria, and similar changes in their derived products trigger and facilitate α-synuclein fibril formation, propagation, and disease pathology (Figure 2).

The genus *Akkermansia* is consistently enriched in human PD patients versus healthy controls, and the data corroborate recent findings in an A53T α-synuclein Tg non-human primate model. Investigators noted a higher diversity of microbial flora with significant increases in abundance of *Akkermansia*, *Sybergistetes*, and *Eggerthella lenta* (Nishiwaki et al., 2020; Yan et al., 2021). As a mucin-degrading bacteria, *Akkermansia* spp. are utilized as a microbial biomarker for determining failure in bacteriotherapy for treating colitis and are implicated in the progression of PD and AD pathology by promoting a pro-inflammatory state (Ihekweazu et al., 2019; Dodiya et al., 2020). While a definitive characterization of *Akkermansia* spp. in neurodegenerative disorders is yet to be elucidated, recent studies in the chronically stressed rotenone-induced PD model indicate elevated abundances of *Akkermansia* spp. could modulate fibril formation and exacerbate α-synuclein expression in the colon (Dodiya et al., 2020). Furthermore, an unpublished report suggests that extracellular mucin modulates the release of the *Akkermansia* protein secretome, which dysregulates calcium homeostasis, leading to the promotion of calcium uptake in the mitochondria of entero-endocrine cells, triggering the formation of reactive oxygen species and subsequent α-synuclein phosphorylation, aggregation, and deposition *in vitro* (Amorim Neto et al., 2021). Although these results are limited to *in vitro* evaluation, it highlights previously unknown functions of distinct microbes in influencing the formation of pathogenic fibrils (Figure 2A). Furthermore, it is conceivable that an already altered microbial community structure in PD, causing an overabundance of pathogenic microbes and a reduction in resident protective microbes, could facilitate α-synuclein aggregation and a PD disease phenotype. A recent strain-level meta analysis of gut microbiota of PD patients supports this notion as a reduced abundance of *Bacteroides ovatus* was noted. *B. ovatus* is a resident protective microbe that converts dietary flavanols to phenolic acids. Phenolic acids such as 3-hydroxybenzoic acid (3-HBA), 3,4-dihydroxybenzoic acid (3,4-diHBA), and 3-(3-hydroxyphenyl)propionic acid (3-HPPA), produced as a consequence of microbial fermentation, interfere with the assembly of monomeric forms of α-synuclein into its protofibrils *in vitro*, and upon administration, improved locomotor activity in an A53T Drosophila model of synucleinopathy (Ho et al., 2019). Additionally, *Bacillus subtilis*, a commensal microbe, can inhibit the aggregation of α-synuclein in both young and aged *Caenorhabditis elegans* models of synucleinopathy in varied pathways (Goya et al., 2020). *B. subtilis* induced biofilm formation (mediated from matrix protein TasA), produced nitric oxide, and upregulated numerous protective pathways, including the sphingolipid metabolic pathway responsible for inhibiting and reversing α-synuclein aggregation (Goya et al., 2020). Adult roundworms that fed on vegetative *B. subtilis* activate downstream transcriptional factors DAF-16/FOXO in the insulin-like receptor DAF-2 pathway to reduce α-synuclein aggregation. Familial PD caused by a mutation in glucocerebrosidase (*GBA1*) leads to the metabolic dysregulation of the important bioactive lipid ceramide and imbalance of its intermediate glucosylceramide (Plotegher et al., 2019). *B. subtilis* was found to further exhibit anti-aggregation properties by regulating the sphingolipid metabolic pathway involving upregulation of LAGR-1/CERS1 (ceramide synthase), ASM-3/SMPD1 (acid sphingomyelinase), and downregulation of SPTL-3/SPTLC2 (serine palmitoyltransferase), therefore, reducing glucosylceramide (Goya et al., 2020). Overall, these studies highlight the dynamic abundances of pathogenic and protective microbes and their metabolic products in propagating or limiting α-synuclein aggregation.

In PD and AD individuals, an increased abundance of Enterobacteriaceae taxa, particularly *Escherichia coli*, is associated with increased concentrations of LPS and the bacterial amyloid protein curli (Scheperjans et al., 2015; Li et al., 2019; Sampson et al., 2020). LPS is a major component of the cell wall of Gram-negative bacteria and is the most well-characterized and understood endotoxin that triggers chronic inflammation and neurodegeneration (Qin et al., 2007), which is found in high concentrations in the blood, gut, skin, and gums during bacterial infections. Several studies show that LPS promotes the aggregation of peripheral amyloid, tau, and α-synuclein, thereby accelerating fibrillogenesis (Lee et al., 2008; Brown, 2019). Notably, NMR studies reveal that α-synuclein aggregation kinetics depends on direct heteromolecular interaction with the LPS-structural motif, leading to downstream nucleation events and stable fibril forms (Figure 2B) (Bhattacharyya et al., 2019). Furthermore, the bacterial curli protein is endogenously produced from microbial species (*E. coli*/*Salmonella* spp.) as they contain the *csgA/B* operon for expression of CsgA/B for biofilm formation. Biofilm formation enables commensal *E. coli* to persist in the colon’s outer mucus layer. However, other strains of *E. coli* that are more invasive, aggregative, pathogenic, hemorrhagic and toxigenic can adhere to the intestinal epithelium and trigger an immune response (Rossi et al., 2018). As curli protein is one of the major components of the bacterial extracellular matrix, it was shown to accelerate fibrilization by cross-seeding and aggregation of α-synuclein and β-amyloid (Ivanova et al., 2021). Although the trigger that contributes to fibrillization and pathological cross-seeding curli protein is unclear, a genome-wide screening analysis identified that bacterial curli amyloid was the distinguishing factor for prompting cross-seed α-synuclein aggregation and dysfunction of mitochondrial cellular respiration in both an *in vitro* neuroblastoma cell line and *in vivo* A53T α-synuclein over-expressing *C. elegans* model (Sampson et al., 2020; Wang et al., 2021). These findings were further confirmed in a Thy1-ASO mouse model, where curli-producing bacteria (*E. coli* strain MC4100) amplified α-synuclein neuropathology in the midbrain and exacerbated motor and GI deficits (Sampson et al., 2020). Bacterial native amyloid monomeric CsgA protein augmented aggregation of α-synuclein proto-fibrils but did not trigger the acceleration of α-synuclein monomer to its oligomeric form, as no interaction occurred between CsgA and α-synuclein monomer *in vitro* (Sampson et al., 2020). Possibly, other transient mechanisms, e.g., CsgA and α-synuclein interaction with Toll-like receptor-2 (Tukel et al., 2009) are involved in triggering either CsgA or α-synuclein oligomerization (Sampson et al., 2020). Although additional studies are warranted, the curli protein is also known to promote aggregation and neurodegeneration of amyloid-β, SOD1-G85R, and huntingtin in AD, ALS, and Huntington disease models, respectively (Wang et al., 2021). Thus, the pro-fibrillogenic status of amyloid curli protein is a functional consequence of altered gut microbial and metabolic composition (Figure 2C).

### Microbial Dysbiosis Modulates Protein Clearance Mechanisms, Autoimmune Functions, and Central Nervous System Immune Recruitment

Aggregated misfolded protein and autophagolysosomal-proteasomal pathways participate in a vicious cycle that causes cytotoxicity and exacerbates hallmark pathologies of neurodegenerative disorders (Monaco and Fraldi, 2020). Previous studies in CNS point to inflammatory mediators as triggers of the dysfunctional autophagy-lysosomal pathway. Recent studies report similar mechanistic pathways contributing to early seeding and formation of peripheral inclusion bodies in the gut. Upon direct intramuscular injection of pre-formed α-synuclein fibrils in the duodenum of ASO mice, investigators report a heightened inflammatory response (IL-6) in the duodenum 7 days post-inoculation (dpi), which likely suggests an early protective response to maintain enteric health (Schafer et al., 1999; Challis et al., 2020). The preliminary inflammatory response was followed by increased fractalkine levels (a marker for macrophage recruitment), heightened levels of macrophage colony-stimulating factor (MCSF, a marker for macrophage recruitment and differentiation), and recruitment of Iba1+ macrophages in a time-dependent manner. Additionally, no changes to myenteric neuronal cell count were observed; however, the neuronal cell volume had significantly decreased seven dpi, which recovered by 21 dpi. On the contrary, the GFAP+ myenteric EGC count and volume showed a sustained increase over time, reflective of reactive gliosis (Challis et al., 2020). Accumulation of pathological α-synuclein aggregates and a heightened state of gliosis indicate impairment of relevant protein clearance pathways (Fellner et al., 2011). Of the several lysosomal proteins, variants of the major lysosomal enzyme GBA1 are implicated with increased PD risk (Atashrazm et al., 2018). α-Synuclein aggregates can directly inhibit GCase activity and set up a futile feed-forward loop to increase both α-synuclein levels and neurotoxicity (Mazzulli et al., 2011). In the gut, α-synuclein fibrils inhibited the GBA1 function, leading to loss of ENS connectivity and GI functional deficits in aged ASO mice, not young mice (Challis et al., 2020). Thus, age-dependent loss of GBA1 function and heightened susceptibility to peripheral α-synuclein pathology could depend on the loss of ‘healthy’ microbes. As discussed earlier, microbes such as *B. subtilis* can reduce α-synuclein load directly by activating the DAF-2 signaling pathway and indirectly by regulating the sphingolipid metabolic pathway (Goya et al., 2020). Since *B. subtilis* abundance is reduced in an age-dependent manner, supporting GBA1 function by microbial supplementation could limit α-synuclein pathology.

**FIGURE 3:**
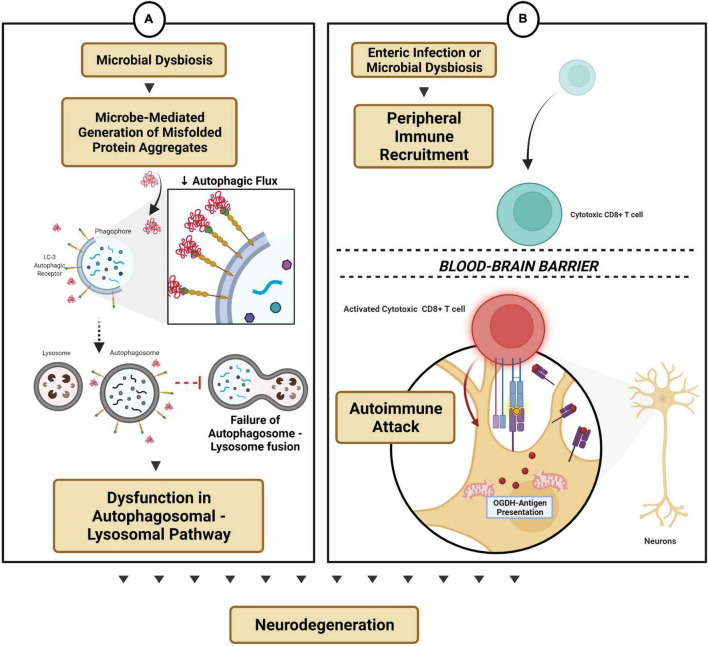
Microbial dysbiosis-modulated protein mechanisms and autoimmune functions. **(A)** Microbe-mediated generation of protein aggregates reduces autophagic flux and thereby dysregulates the autophagosomal-lysosomal pathway. **(B)** Enteric infection triggers peripheral immune recruitment into the CNS by gaining access across the blood–brain barrier, thereby triggering an autoimmune attack on subsets of neurons (e.g., dopaminergic neurons).

Failure of the autophagosomal-proteasomal functions accounts for a substantial percentage of sporadic PD cases (reviewed in Maloney and Lahiri, 2016; Zheng et al., 2016). Interestingly, recent reports suggest that appendectomies lowered PD risk, while individuals with an intact vermiform appendix showed increased levels of truncated soluble α-synuclein, capable of oligomerization and propagation (Killinger et al., 2018). As a vestigial organ, the appendix is indeed a reservoir of beneficial microbes, and it is suggested to be involved in repopulating the large intestine with commensal flora after diarrhea and, in general, maintains intestinal health (Randal Bollinger et al., 2007; Im et al., 2011; Guinane et al., 2013; Sahami et al., 2016). Furthermore, the appendix contains mucosa and submucosa rich with macrophages, follicular dendritic cells, and lymphocytes, which aid in detecting and tolerizing the body to host and foreign antigens (Kooij et al., 2016). But a growing number of studies have identified that the vermiform appendix can also contribute to the development of a chronic intestinal inflammatory state (Sahami et al., 2016). The presence of the appendix significantly correlates with the development of ulcerative colitis, while an inverse association holds for individuals with appendectomies (Sahami et al., 2016). Similar findings were observed in murine colitis models (Mizoguchi et al., 1996; Farkas et al., 2005). Additionally, in analyzing the role of the cecal patch in GF-mice, Masahata et al. (2014) revealed that removal of cecal lymphoid tissue in animals decreased colonic IgA+ cell accumulation in the large intestine and altered fecal microbiota composition. Although a clear mechanistic perspective is lacking, it can be conceptualized that gut microbial dysbiosis (and its metabolites) modulates innate and adaptive immune players by recruiting immune secreting cells from the appendix, thereby priming the immune system for an enhanced inflammatory response and a possible source of α-synuclein misfolding (Masahata et al., 2014; Morrison and Preston, 2016; Zheng et al., 2020b). Although it is well characterized that pathological α-synuclein propagates in a prion-like fashion from the stomach and duodenum to the brain *via* the vagus (Kim et al., 2019; Van Den Berge et al., 2019), how the appendix facilitates the pathological spread is unanswered since the evidence shows increased levels of α-synuclein with significant enrichment within the axonal varicosities of the mucosal plexus (Gray et al., 2014). Furthermore, the pathological forms of truncated α-synuclein were also identified within a healthy human appendix (Killinger et al., 2018). Interestingly, accumulation of α-synuclein was found in intralysosomal sites within appendiceal mucosal CD68+ macrophages (Gordevicius et al., 2021). As mucosal immune cells contain endolysosomal proteins responsible for the breakdown of α-synuclein, deeper investigations into the appendix of PD patients identified epigenetic silencing *via* DNA methylation of genes related to the production of autophagic protein and lysosomal degradation pathways (Gordevicius et al., 2021). Although the factors that promote this dysregulation are poorly understood in PD, recent data suggest that increased α-synuclein levels can reduce autophagic flux by decreasing the autophagosomal membrane-associated protein LC3, resulting in further accumulation of α-synuclein and cytotoxicity (Figure 3A) (Lei et al., 2019). Furthermore, the shift in microbial species triggers gut inflammatory pathways and weakens homeostatic autophagosomal-proteasomal functions (necessary for suppressing inflammation). Deteriorated clearance mechanism leads to the accumulation and sensitization of α-synuclein *in vivo*, thereby increasing the risk of developing PD (Lei et al., 2019). Similarly, the gut microbiota modulate β-amyloid and tau AD pathology. In a study in the 5XFAD mouse model of AD, microbial dysbiosis was noted to be associated with activation of the endolysosomal CCAAT/enhancer-binding protein-β (C/EBP-β) – asparagine endopeptidase (AEP) pathway (Chen et al., 2020). AEP is present in the endolysosome and is implicated in causing aberrant cleaving of amyloid precursor protein (APP N585) and tau N368 leading to plaques and neurofibrillary tangles (NFTs), while C/EBP-β is involved in inducing expression of proinflammatory signaling molecules in microglia and astrocytes and participating in a vicious feedback loop that worsens in an age-dependent manner (Basurto-Islas et al., 2018; Wang et al., 2018). Implanting gut microbiota from aged 3xTg AD mice (APP Swedish, MAPT P301L, and PSEN1 M146V) to young 3xTg mice increased intestinal permeability and temporally augmented C/EBPβ/AEP activation (Chen et al., 2020). These findings support temporal enteric neuronal loss, increased inflammation in the gut, and increased expression of AEP and C/EBPβ in the brain. Interestingly, antibiotic administration diminished the C/EBP-β/AEP signaling pathway in the brain, reduced proinflammatory Iba+ microglia signals, abolished the aggregation of Aβ fibrils measured by thioflavin S assay, and restored cognitive functions (Chen et al., 2020). As these findings do not directly implicate a specific microbial population, age-dependent reductions in diversity and richness of microbial species, with increased gut permeability and infiltration of toxic microbial metabolites such as LPS and amyloids, likely stimulate peripheral C/EBP-β/AEP signaling, and thereby progressive Aβ and NFT pathologies (Chen et al., 2020).

The peripheral etiology of neurodegenerative disorders links the role of gut microbiota in influencing T-cell differentiation and autoimmune regulation. CD4^+^ T-cell subsets, T-helper 17 (Th17), and regulatory T cells (Treg) play a vital role by limiting α-synuclein-mediated microglial activation and reducing the activity of CD8^+^ cytotoxic T-cell and natural killer (NK) cells during the neuroinflammation processes in PD (Chen et al., 2018). Interestingly, CD8^+^ T-cell infiltration in the substantia nigra pars compacta was observed in the brain in early pre-symptomatic PD, which correlated with α-synuclein aggregate accumulation and eventual dopaminergic neuronal cell loss (Galiano-Landeira et al., 2020). Microbial flora plays a crucial role in regulating and differentiating CD4^+^ T-cell subsets (Geuking and Burkhard, 2020). Critical Th17/Treg imbalances have been linked to PD pathogenesis (Chen et al., 2015; Zheng et al., 2020b). Understanding microbiome-mediated immunomodulation arises from studies in *B. fragilis*, an important gut-residing bacterium that expresses a capsular protein, polysaccharide A (PSA). PSA can bind to B-cells, triggering Treg cells to release IL-10 and thereby protect against herpes simplex encephalitis (Sittipo et al., 2018; Ramakrishna et al., 2019). Additionally, PSA protected against pathogenic infections, intestinal inflammatory diseases and triggered anti-inflammatory signaling pathways by plasmacytoid dendritic cells and the Toll-like receptor-2-mediated CD9 signaling mechanism (Erturk-Hasdemir et al., 2019). A longitudinal Japanese PD cohort study reported a gradual reduction in the absolute abundances of *B. fragilis* quantified by targeted rRNA qRT-PCR (Minato et al., 2017; Zheng et al., 2020a). Although limited by sample size, the findings suggest that intestinal dysbiosis can trigger an autoimmune response and participate in the pathogenesis of PD. Indeed, Pink1-KO mice, a model that is typically asymptomatic of PD and void of dopaminergic degeneration, exhibited a pronounced reduction in the density of tyrosine hydroxylase-positive neurons in the dorsal and ventral striatum and dopamine transporters following infection by a pathogenic Gram-negative bacterium, *Citrobacter rodentium* (Matheoud et al., 2019). Mutation within the genes of *PINK1* and *PRKN* ubiquitin ligase impairs the process of mitophagy and is associated with the early onset of PD (Ge et al., 2020). Infection by the mouse intestinal pathogen *C. rodentium* exclusively led to the increased presentation of mitochondrial antigen protein 2-oxoglutarate dehydrogenase (OGDH) on its matrix, expression of the MHC class I molecule on the surface of dopaminergic neurons, infiltration of mitochondrial specific CD8^+^ T-cells into the brain, dopaminergic neuronal dysfunction and motor impairment (Figure 3B) (Matheoud et al., 2019; Quinn et al., 2020). A follow-up investigation reported that microbial diversity post-infection by *C. rodentium* was similar in both Pink1-KO mice and their littermate controls, however, the immune activation response was varied (Cannon et al., 2020). At the peak of infection, the level of butyric acid, an SCFA, was disproportionally increased in Pink1-KO mice, possibly implying a compensatory response in early-stage PD pathology (Cannon et al., 2020). Nevertheless, a more detailed mechanistic evaluation is necessary to understand the genetic and environmental roles of microbial immunomodulation and adaptive immunity in the etiology of PD.

As mentioned, a pro-inflammatory state is required to recruit peripheral immune cells into the brain parenchyma, and dysregulation within this process can promote neurodegeneration (Scheld et al., 2016). Since the gut is a gateway for environmental pathogens to enter, and the CNS and ENS participate in pathogen resistance mechanisms, the microbial flora and their metabolites are essential for promoting tight-junction expression and maintaining blood–brain barrier (BBB) function (Braniste et al., 2014). Indeed, microbial dysbiosis, induced by enterobacterial infection, enabled peripheral immune hemocyte recruitment by triggering brain-reactive oxidative species and aggravating neurodegeneration in a *Drosophila* model of AD (Wu et al., 2017). Furthermore, since T-cells are actively recruited into the brain during a pro-inflammatory response, astrocytes modulate the anti-inflammatory response by activating the TNF-related apoptosis-inducing ligand (TRAIL) (Sanmarco et al., 2021). As TRAIL-expressing astrocytes have been previously demonstrated to induce T-cell apoptosis and downstream CNS inflammation, notably, the expression of this receptor was dependent on meningeal IFNγ expression from NK cells, and the microbiota was known to induce IFNγ expression from NK cells (Burgaletto et al., 2020; Sanmarco et al., 2021). Enteric infection decreases TRAIL expression, thereby promoting subsets of astrocytes to become pathogenic in experimental autoimmune encephalomyelitis, a model for multiple sclerosis (Sanmarco et al., 2021). NK cells are typically prevented from crossing the BBB; however, T-cells can alter their phenotype for increased expression of cell adhesion molecules, chemokine and cytokine receptors, and matrix-degrading enzymes for BBB attachment-gaining entrance (Flugel et al., 2001). Thus, normobiosis within the microbiota enables a basal homeostatic physiological immune response by modulating immune effector sites in the CNS. Bacterial infection or microbial dysbiosis can limit immunoregulatory activity in neuroglial cells, thereby exacerbating neuroinflammation and neurodegeneration. Beyond peripheral immune recruitment, microbial-derived SCFAs and tryptophan metabolites readily gain access through the BBB and into the CNS and act directly on astrocytes and microglia *via* AHR, SCFAs, and the FFAR2 receptor, respectively, for modulating maturation, function, and inflammation (Braniste et al., 2014; Erny et al., 2015; Rothhammer et al., 2016, 2018; Powell et al., 2017; Wekerle, 2017).

## Conclusion

To summarize, the microbiota is a vital modulator for several disease etiologies, including neurodegenerative diseases. As correlative studies in large-cohort populations provide a global view, mechanistic elucidation permits the identification of well-defined molecular pathways that have immense implications for early biomarker discovery and the development of novel therapies. This review attempts to provide some mechanistic insight (Figures 1–3) into how the bi-directional network between the gut microbiota and the brain can not only protect but also trigger and aid chronic gut inflammation, promote a global pro-inflammatory state, modify and contribute to protein misfolding processes, dysregulate the autophagic-lysosomal protein clearance mechanism, differentiate T-cell subsets to facilitate a deleterious autoimmune response, and finally modulate immune recruitment and BBB status. We also discussed how the gut microbiota and the ENS maintain gut physiology and limit enteric neuropathy by CNS-mediated circuits and neuro-immune synapses. As this field is rapidly evolving, and a growing number of studies support the notion that alterations in the microbiome and disease-specific pathologies occur several years before the onset of neurodegeneration, a more refined diagnostic procedure and development of novel intervention strategies (e.g., microbiome-based therapeutics) targeting the microbiome will greatly benefit translational discovery efforts in neurodegeneration.

## Author Contributions

PP conceived and wrote the manuscript with the help of CW and AGK. KS, GZ, HJ, VA, and AK edited and provided critical feedback for approval of the final manuscript. AGK supervised, conceived the topics with PP, and extended crucial input to support the final manuscript. All authors contributed to the article and approved the submitted version.

## Conflict of Interest

AGK has an equity interest in PK Biosciences Corporation and Probiome Therapeutics located in Ames, IA, United States. The terms of this arrangement have been reviewed and approved by Iowa State University and University of Georgia in accordance with its conflict-of-interest policies. The remaining authors declare that the research was conducted in the absence of any commercial or financial relationships that could be construed as a potential conflict of interest.

## Publisher’s Note

All claims expressed in this article are solely those of the authors and do not necessarily represent those of their affiliated organizations, or those of the publisher, the editors and the reviewers. Any product that may be evaluated in this article, or claim that may be made by its manufacturer, is not guaranteed or endorsed by the publisher.
